# Pediatric to adult healthcare transitioning for adolescents living with HIV in Nigeria: A national survey

**DOI:** 10.1371/journal.pone.0198802

**Published:** 2018-06-12

**Authors:** Okikiolu A. Badejo, William N. A. Menson, Nadia A. Sam-Agudu, Jennifer Pharr, Salome Erekaha, Tamara Bruno, Gift Nwanne, Olabanjo Ogunsola, Jude Ilozumba, Olusegun Busari, Echezona E. Ezeanolue

**Affiliations:** 1 FHI360, Abuja, Nigeria; 2 Global Health Initiative, School of Community Health Sciences, University of Nevada, Las Vegas, Nevada, United States of America; 3 International Research Center of Excellence, Institute of Human Virology Nigeria, Abuja, Nigeria; 4 Institute of Human Virology, University of Maryland School of Medicine, Baltimore, Maryland, United States of America; 5 APIN Public Health Initiatives, Abuja, Nigeria; 6 Center for Clinical Care and Clinical Research Nigeria, Abuja, Nigeria; 7 Center for Integrated Health Programs, Abuja, Nigeria; 8 HealthySunrise Foundation, Las Vegas, Nevada, United States of America; 9 Department of Paediatrics and Child Health, University of Nigeria Nsukka, Enugu, Nigeria; The Ohio State University, UNITED STATES

## Abstract

**Introduction:**

The period of transition from pediatric to adult care has been associated with poor health outcomes among 10–19 year old adolescents living with HIV (ALHIV). This has prompted a focus on the quality of transition services, especially in high ALHIV-burden countries. Due to lack of guidelines, there are no healthcare transition standards for Nigeria’s estimated 240,000 ALHIV. We conducted a nationwide survey to characterize routine transition procedures for Nigerian ALHIV.

**Materials and methods:**

This cross-sectional survey was conducted at public healthcare facilities supported by five local HIV service implementing partners. Comprehensive HIV treatment facilities with ≥1 year of HIV service provision and ≥20 ALHIVs enrolled were selected. A structured questionnaire assessed availability of treatment, care and transition services for ALHIV. Transition was defined as a preparatory process catering to the medical, psychosocial, and educational needs of adolescents moving from pediatric to adult care. Comprehensive transition services were defined by 6 core elements: policy, tracking and monitoring, readiness evaluation, planning, transfer of care, and follow-up.

**Results:**

All 152 eligible facilities were surveyed and comprised 106 (69.7%) secondary and 46 (30.3%) tertiary centers at which 17,662 ALHIV were enrolled. The majority (73, 48.3%) of the 151 facilities responding to the “clinic type” question were family-centered and saw all clients together regardless of age. Only 42 (27.8%) facilities had an adolescent-specific HIV clinic; 53 (35.1%) had separate pediatric/adolescent and adult HIV clinics, of which 39 (73.6%) reported having a transfer/transition policy. Only 6 (15.4%) of these 39 facilities reported having a written protocol. There was a bimodal peak at 15 and 18 years for age of ALHIV transfer to adult care. No surveyed facility met the study definition for comprehensive transition services.

**Conclusions:**

Facilities surveyed were more likely to have non-specialized HIV treatment services and had loosely-defined, abrupt transfer versus transition practices, which lacked the core transition elements. Evidence-based standards of transitional care tailored to non-specialized HIV treatment programs need to be established to optimize transition outcomes among ALHIV in Nigeria and in similar settings.

## Introduction

Despite a 30% decrease in the number of all AIDS-related deaths between 2005 and 2012, mortality among adolescents living with HIV (ALHIV) increased by 50% within that same period [1,2]. Nearly 92% of these deaths occurred in sub-Saharan Africa [3], where AIDS is the leading cause of mortality among adolescents [4,5]. This has been attributed in part to high loss to follow-up (LTFU) rates that appear to spike even higher around the period of transition from pediatric to adult care [6,7]. Multiple studies on HIV and other chronic illnesses among adolescents have demonstrated high drop-out rates around the transition period, leading to poor retention in care and increased morbidity and mortality [6,8–11].

Healthcare transition is defined as “the purposeful, planned movement of adolescents and young adults with chronic physical and medical conditions from child-centered to adult-oriented health care systems” [10]. Adolescent transition practices vary across different settings and disease conditions. Studies on transition models have to date been largely conducted in resource-rich North American and European settings among adolescents and young people with various chronic health conditions including diabetes, cystic fibrosis, and organ transplantation [12–14]. The few available studies evaluating transition models among adolescents living with HIV (ALHIV) were also conducted largely in North America and Europe, and engaged patients between 15 and 23 years of age [15–17]. Education programs, transition coordinators and interdisciplinary teams, transition readiness evaluation, visits to both pediatric and adult clinics during transition, and enhanced follow-up were identified by these studies as interventions and strategies that impacted positively on transition outcomes [16,17].

There is a lack of data on prevailing transition practices and limited evidence for effective transition strategies for ALHIV in Africa [17]. A recent review on the needs and challenges of transitioning ALHIV in sub-Saharan Africa noted that most African countries had no specific national guidelines on when and how to transition ALHIV to adult care [18]. Nigerian ALHIV account for approximately 15% of the 1.75 million ALHIV in sub-Saharan Africa, second only to South Africa [19,20]_._ The large number of Nigerian ALHIV require comprehensive transition services to usher them into adult care with minimized drop-out, morbidity and mortality. It is concerning to note that among the 5 countries with the highest burdens of ALHIV (South Africa, Nigeria, Kenya, Tanzania and Uganda), only Nigeria continues to experience increases in mortality among both younger and older adolescents [21]. Nigeria’s Federal Ministry of Health has developed multiple policies regarding adolescent and young people’s health, including HIV care and treatment [22–24]. However, these documents and the current national HIV guidelines [25] provide little guidance on transitioning from pediatric to adult HIV care. Due to the lack of transition guidelines in Nigeria, this process is likely to vary widely across healthcare facilities.

The purpose of this study was to characterize current practices and services available for transitioning ALHIV from pediatric to adult HIV care across Nigeria.

## Methods

### Study design and setting

This cross-sectional survey was a collaboration between five Nigerian non-governmental organizations (NGOs) that serve as in-country implementing partners for the United States’ Presidency Emergency Plan for AIDS Relief (PEPFAR): APIN Public Health Initiatives; Center for Clinical Care and Clinical Research, Nigeria; Center for Integrated Health Programs, FHI 360, and the Institute of Human Virology Nigeria. Within our study setting in Nigeria, between 75 and 82% of annual HIV service financing has been supported by external donors to date, the two largest being PEPFAR and the Global Fund [26]. However, PEPFAR support has singularly encompassed up to 65% of annual expenditures for Nigeria’s HIV program [26–28].

All five NGOs participating in this study are institutional members of the Nigeria Implementation Science Alliance, an umbrella organization established to facilitate the generation and application of impactful evidence towards health improvements in Nigeria [24]. With PEPFAR funding, these five NGOs provide support for HIV prevention, treatment and care at nearly 1,000 comprehensive HIV treatment centers across 30 of the 37 states and territories in Nigeria. At the time of the survey, these HIV treatment centers catered to over 24,000 Nigerian ALHIV aged 10 to 19 years. The survey was conducted as a program evaluation as well as pre-study site assessments to inform the implementation of the Adolescent Coordinated Transition (ACT) study, a National Institutes of Health-funded study on adolescent HIV transitioning in Nigeria [29].

### Study site selection criteria

PEPFAR-supported sites in Nigeria receive HIV-related service funding and technical support from PEPFAR-funded NGOs known as implementing partners. Operations and commodities for services including HIV testing, early infant diagnosis, CD4/viral load monitoring, anti-retroviral drugs and drugs for HIV co-infections/opportunistic infections, programmatic data collection, healthcare worker capacity-building and program quality improvements are financed and/or facilitated by the implementing partner with PEPFAR funds. Additionally, implementing partners train and provide supportive supervision and mentoring to government-employed healthcare workers to provide competent prevention, care and treatment for clients per existing national guidelines. At each facility, one healthcare worker functions as the focal person for each pediatric/adolescent, adult, and prevention of mother-to-child transmission of HIV (PMTCT) program. It should be noted that depending on the level of care (primary, secondary or tertiary) and human resources for health available, facilities may provide services for only one programmatic area-for example PMTCT services only, or all of them.

Sites were selected from the network of facilities supported by the five participating implementing partners, based on the following criteria:

- PEPFAR-supported public healthcare facility
- Provision of comprehensive HIV services (HIV prevention, care and treatment services to adults, adolescents, children and pregnant women) for ≥1 year
- ≥ 20 ALHIV aged 10 to 19 years enrolled in care as of July 31, 2016.

Primary healthcare facilities were excluded as these sites were not mandated to provide pediatric HIV treatment services per national guidelines [30]; however these sites may have older adolescents enrolled in adult care.

The selection strategy focused on facilities experienced in HIV care that had received sufficient capacity-building and mentoring to establish all components of HIV service delivery according to national HIV guidelines. Furthermore, the requirement of at least 20 ALHIV enrolled allowed for surveying of sites that had a consistently sizeable cohort of adolescents with which to maintain a sustained pediatric/adolescent HIV program. PEPFAR-only sites were selected because PEPFAR funding was common to all the participating NGOs; not all participating NGOs received Global Fund support. Additionally, the geographical scope of PEPFAR support provided by these five implementing partners covered all six geopolitical zones, ensuring national representation towards generalizable findings.

### Ethical considerations

This survey was conducted as pre-study site assessments and a program evaluation that did not involve patient contact, the viewing of medical records nor the collection of names, addresses or any other identifying patient data from adolescents enrolled at the study facilities. The leadership for each NGO as well as focal persons at each surveyed site were briefed on the study procedures and nature of data to be collected before study initiation. The lead NGO for this study, the Institute of Human Virology Nigeria, obtained prior approval from the Nigerian National Health Research Ethics Committee for the analysis of HIV programmatic data for program evaluation. Separate ethical approval was obtained for the prospective ACT study.

### Study tool and data collection

For this study, we use the term ‘transfer’ to refer to an often abrupt and passive change in healthcare provider and/or clinic for adolescents moving from pediatric to adult care with little or no preparation or follow up [31]. We use ‘transition’ to refer to an active process that prepares and caters to the medical, psychosocial, and educational needs of adolescents as they get ready to move from pediatric to adult-oriented care [31].

Both study and HIV program staff developed the structured survey questionnaire [S1 File], which was based on the Got Transition Center’s 6 core elements of healthcare transition of youth into adult care [32], as follows:

- A Transition Policy: Developing a transition policy/statement with input from adolescents and families that describes the practice’s approach to transition.
- Transition Tracking and Monitoring: Establish criteria and process for identifying transitioning youth
- Assessment of Transition Readiness: Conduct regular transition readiness assessments
- Transition Planning: Plan with youth/parent/caregiver for optimal timing of transfer
- Transfer of Care: Confirm date of first adult provider appointment; Transfer young adult when his/her condition is stable, complete transfer package, including medical summary.
- Verification of Transfer Completion: Contact young adult and parent/caregiver a few months after last pediatric visit to confirm transfer of responsibilities to adult practice; communicate with adult practice confirming completion of transfer.

The Got Transition Center’s elements for healthcare transitioning are in line with transitioning guidelines endorsed by pediatric, family practice, and internal medicine societies in the United States[32], as well as being consistent with the World Health Organization’s recommendations for transitioning of ALHIV [33].

The survey questionnaire was ultimately organized into five sections:

**Section A**: *Site profile*: brief description of the facility and aggregate ALHIV data.

**Section B**: *Clinic services*: information on services provided to pediatric and adolescent clients.

**Section C**: *Adolescent care services*: described services provided in the adolescent clinic, if any existed at the surveyed facility.

**Section D**: *Adolescent support group services* was used to ascertain support group activities available for ALHIV.

**Section E**: *Transfer of adolescents from pediatric to adult clinic*: described routine practices of transfer/transition from the pediatric to adult clinic.

The survey questionnaire was administered to focal persons for the adult, adolescent and/or pediatric HIV programs who provided relevant service availability/delivery data regarding the study sites. Where these focal persons were not appointed or not available, other facility staff with relevant knowledge of pediatric and adolescent HIV care at their facility were interviewed. The questionnaire was administered in person by trained study staff from each of the five implementing partners. Phone interviews were conducted for facilities where respondents were physically unavailable but offered to provide information. Data on ALHIV enrollments were abstracted from routine programmatic reporting tools that collected only aggregated data.

All data was collected manually on hard copy questionnaire forms and entered by study coordinators from the 5 implementing partners into a Microsoft Access database. Second and third-level validations were conducted by the central study team, using the completed hard copies against entries in the electronic Microsoft Access database. Descriptive statistics were applied to the data; in addition, a two-sample t-test was conducted to compare the mean age of transfer between facilities that had an ALHIV clinic and those that did not. Data were analyzed using SPSS version 21 [34]. The survey was initiated and completed in September 2016.

## Results

All facilities that met the predefined selection criteria were surveyed. A total of 152 comprehensive healthcare facilities were included, and had a total of 17,662 ALHIV enrolled comprising 7,814 young ALHIV 10–14 years old and 9,848 older ALHIV 15–19 years old. ALHIV enrollment ranged between 20 and 348 per surveyed facility. Surveyed facilities spanned a 30-state network of sites supported by the five implementing partners in all six geopolitical zones of Nigeria. Of the 152 facilities surveyed, 106 (69.7%) were secondary-level and 46 (30.3%) were tertiary healthcare centers. Most (67.8%) of the 180 survey respondents were doctors, followed by an equal proportion of nurses (16.1%) and other healthcare worker cadres (16.1%, comprising largely of monitoring and evaluation officers and adherence counsellors). Nearly three-quarters (72.8%) of all respondents were serving as pediatric, adult or general ART focal persons at their facilities.

Out of a total of 151 facilities (one missing response), 73 (48.3%) saw all HIV-positive clients together regardless of age in family-centered clinics; 42 (27.8%) saw ALHIV in separate adolescent clinics, and 36 facilities (23.8%) saw younger and older adolescent clients in pediatric and adult clinics respectively. Approximately 88% of facilities (133/151, with one missing response) had been providing adult HIV services for six years or more, while 85% (128/151) had been providing pediatric HIV services for six years and more. An additional 16/151 (10.6%) and 19/151 (12.6%) facilities reported delivering adult and pediatric HIV services for 4–6 years respectively. Only two facilities reported providing adult or pediatric HIV services for 1–3 years, and another two reported providing pediatric services for less than one year.

Fifty-three (34.9%) of 151 facilities had separate pediatric/adolescent and adult HIV clinics. Thirty-nine (73.6%) of these 53 facilities reported having an established practice for transferring/transitioning adolescents from pediatric to adult care; however, only six of these had a written protocol (Table 1). All 39 facilities that reported having a routine practice for transferring/transitioning ALHIV used age as a transfer criterion. Only 37/152 (24.3%) facilities had established ALHIV support groups (Table 1).

**Table 1:** Services available and routine practices for transferring/transitioning alhiv from pediatric to adult care.

	No. of Facilities (N = 152)	%
ALHIV Clinic^a^	42	27.8
ALHIV Support Group	37	24.3
**Separate Adult and Pediatric/ALHIV Clinics**	**N = 53**	%
Tertiary level of care	35	66.0
Secondary level of care	18	34.0
Routine practice for ALHIV transfer/transition^b^	39	73.6
Written transfer/transition protocol^b^	6	11.3
Pediatric-adult clinic communication during transfer/transition^b^	36	67.9
**Method of pediatric-adult clinic communication during transfer/transition**	N = 36%	
Verbal only	6	16.7
Written only	16	44.4
Verbal + Written	14	38.9
**Post-transfer follow-up by pediatric clinic**	**N = 12**	
**Method of follow-up**		
Contact parent/ALHIV	9	75.0
Contact adult clinic	3	25.0
Contact parent/ALHIV + adult clinic	0	0.0
**Transfer Criteria^b^^,^^c^**	**N = 39**	
Age	39	100.0
Aware of diagnosis	12	30.8
Readiness assessment	10	25.6
HIV knowledge	9	23.1
Pregnancy	7	17.9
Marriage	6	15.4
CD4 count	3	7.7
Viral Load	1	2.6

^**a**^Out of 151 facilities responding to this survey question

^b^These responses are not mutually exclusive

^c^Out of 53 facilities with separate adult and pediatric clinics

**Fig 1:**
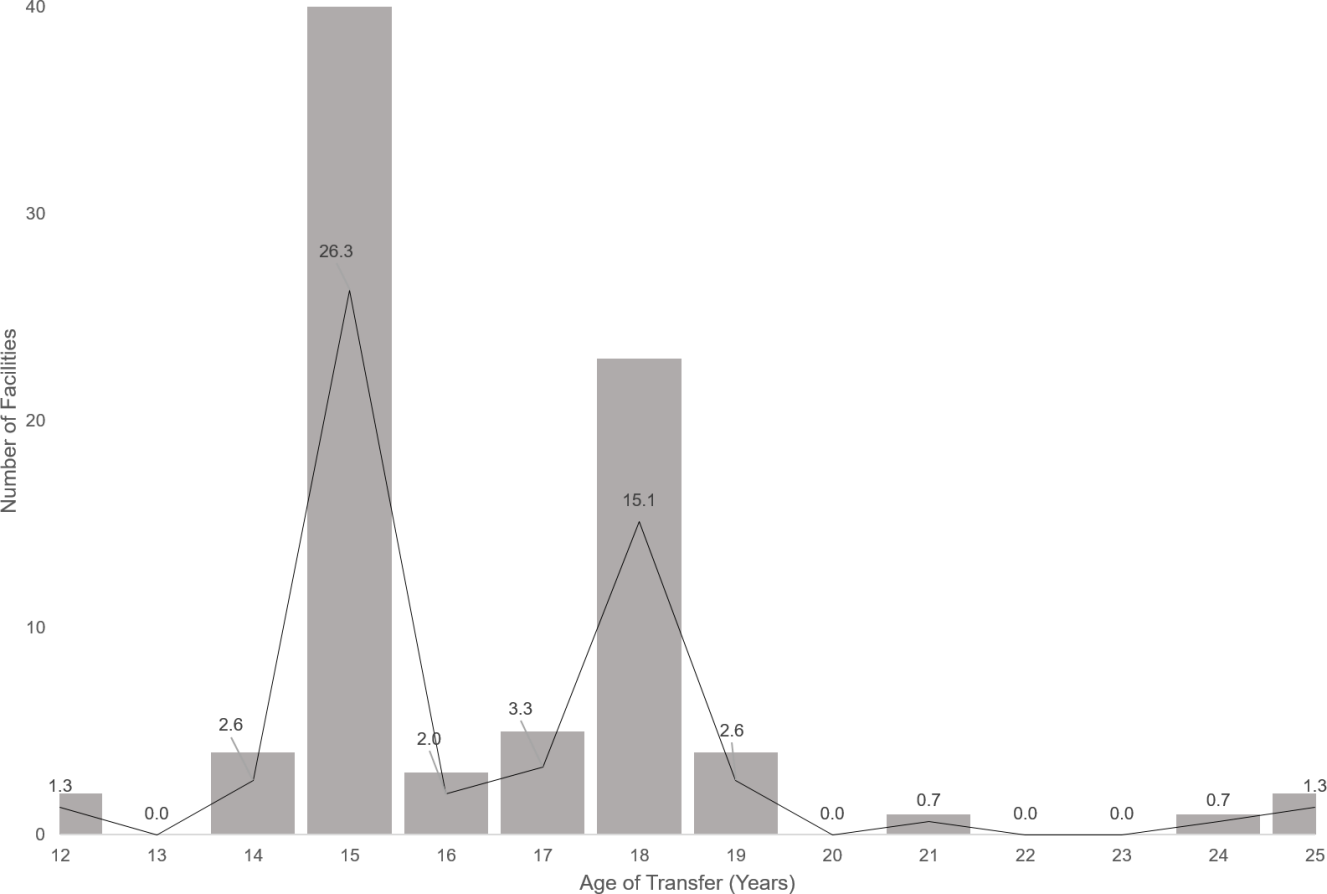
Routine age of alhiv transfer to adult care at surveyed healthcare facilities (N = 85). Plotted lines on bars indicate proportion of facilities reporting corresponding age as their routine age of transfer.

A total of 85 (55.9%) of the 152 surveyed facilities reported having a specific age for ALHIV transfer. Age of ALHIV transfer ranged from 12 to 25 years; however, we observed a bimodal peak in transfer age at 15 years for 40/85 facilities (47.1%) and 18 years for 23/85 facilities (27.1%) (Fig 1). Additionally, among the 85 facilities that responded, the mean age of transfer at 42 facilities that had a dedicated ALHIV clinic was significantly higher (17.4 years) than the 43 facilities that did not have an ALHIV clinic (15.9 years), p = 0.006.

Overall, 72/148 (48.6%) facilities indicated having routine practices for providers to facilitate HIV status disclosure to ALHIV. The median age at which the disclosure process was initiated was 10 years (IQR 8 to 15 years); the minimum-maximum age range was 5 to 18 years. Only 11/72 (15.3%) facilities reported having a written disclosure protocol.

Cadre and numbers of healthcare workers delivering pediatric and adult HIV services at surveyed facilities are displayed in Table 2. Secondary facilities had a noticeable absence of doctors with regards to pediatric HIV clinics. Overall, psychosocial staff numbers were relatively sparse across all clinics and facilities.

**Table 2:** Healthcare worker strength and cadre at pediatric and adult HIV clinics^a^.

	Doctors	Nurses^b^	Pharmacy staff^c^	Psychosocial staff^d^
**Tertiary Facilities (N = 46)**	**7 (5–15)**	**7 (6–10)**	**1 (0–3)**	**2 (0–5)**
**Pediatric HIV clinics**	3 (1–5)	2 (0–6)	0 (0–0)	0 (0–0)
**Adult HIV clinics**	5 (4–10)	5 (4–8)	1 (0–3)	0 (0–4)
**Secondary Facilities (N = 106)^e^**	**3 (1–4)**	**4 (2–8)**	**1 (0–3)**	**0 (0–2)**
**Pediatric HIV clinics**	0 (0–0)	0 (0–0)	0 (0–0)	0 (0–0)
**Adult HIV clinics**	2 (1–3)	3 (2–6)	1 (0–2)	0 (0–2)
**All facilities (N = 152)**	**4 (2–7)**	**6 (3–8)**	**1 (0–3)**	**0 (0–3)**

^a^ All data displayed as median (interquartile range) number of healthcare workers per clinic

^b^ Includes nurse-midwives

^c^ Includes pharmacy attendants, technicians and pharmacists

^d^ Includes adherence counselors, social workers, psychologists, peer educators/treatment supporters, volunteer PLHIVs and mentor mothers

^e^ Includes facilities with separate as well as non-differentiated family-centered HIV clinics. Thus “pediatric” clinics with a median of 0 healthcare workers indicated include the family-centered majority who have the adult care-oriented providers for all HIV client populations.

## Discussion

Among facilities surveyed across Nigeria, our study found routine practices bearing similarities to the major models of adolescent transition/transfer described in the literature [29]: transition between distinct pediatric and adult HIV providers and services; transition between the same providers/clinic with or without a family-centered approach, and transition utilizing adolescent transition clinics.

Approximately 35% of surveyed facilities, mostly tertiary-level, operated separate pediatric and adult HIV clinics. Even though nearly 75% of these mostly specialized facilities reported having an established ALHIV transition policy, none of them, and indeed none of the 152 surveyed facilities, fulfilled all of the Got Transition Center’s six core elements of healthcare transitioning for youth [32]. Abrupt transfer focusing largely on age- rather than a transition process- was the common practice among 39 surveyed facilities that reported having an established “transition” policy. The paucity of written protocols highlights the lack of well-established, evidence-informed guidelines for adoption and use at the facility level. Our findings are consistent with what prevails in many other sub-Saharan African countries [35–38] explained in part by the lack of clinic infrastructure and adolescent health-trained staff [35]. However, transition has been noted to be a somewhat newer concept particularly in Nigeria’s sub-region of West and Central Africa, in part due to the predominance of family-centered care models in this region’s public health sector [35].

Of equal importance are transition practices in non-specialized settings, which were the prevailing circumstances at two-thirds of all surveyed sites. This is not surprising, given the predominance of non-specialized care in sub-Saharan Africa [33]. Age was the most common criterion for transfer across surveyed sites, including at non-specialized facilities which did not have separate pediatric and adult clinics. Reported transfers at these sites are likely due to external transfers to other facilities, or more so, “programmatic transfers”- where adolescents reaching the reporting age for adults in the HIV program (15 years and above) are re-categorized as adults for reporting purposes. This would explain the observed peak at 15 years for transfer; the second peak at age 18 years is most likely due to the attainment of legal age and/or delayed ALHIV transfers to adult clinics at facilities with established adolescent HIV clinics. While chronological age appears to be a strong transfer criterion, it is also critical to consider readiness by age of maturity of the patient, which may vary depending on level of social support, education, and learning capabilities[35,36].

Whatever the case, ALHIV transition practice in non-specialized, more family-centered settings deserve more attention given their predominance, as demonstrated by our study. Adolescent HIV clinics were in existence at less than a third of surveyed sites, and facilities with these clinics are often specialized. Although one study [39] showed relatively higher retention in care among adolescents in a non-specialized and family-centered care program, outcome results of different care models for transitioned/transferred adolescents in Africa have been few and mixed [40–42]. Clearly, transitioning ALHIVs and the challenges they face are heterogeneous, and more studies are needed to further describe their characteristics and needs. However, this also raises the challenge of establishing differentiated care models in non-specialized settings, which is a particular challenge in West and Central Africa [38]. The Got Transition Center has additional guidelines for transitioning adolescents to an adult approach to healthcare within non-specialized settings where pediatric and adult care clinics are not physically separate [32]. This requires the family/general health provider to change their approach to care for the transitioning adolescent, which includes gradually integrating the latter into adult model of care. This approach to transition appears much better-suited to the family-centered care model that exists across much of Nigeria and in similar settings.

Pregnancy and marriage were also reported as criteria for transitioning adolescents at some of our surveyed facilities. While these events may appear to be natural indicators for adulthood and therefore adult care, some pregnant and married ALHIV may be still too young or not mature enough to be transferred to adult care. This may be especially true in some parts of Nigeria where early marriage is not uncommon, especially among adolescent girls. The combination of teenage pregnancy (within or outside the context of marriage) and HIV infection may require more support and a prolonged transition period before transfer to adult care. These adolescents may be at higher risk of LTFU and may subsequently develop poor clinical, physical and psychosocial outcomes if transition is not successful [6]. Additionally, PMTCT outcomes are consistently reported to be worse for adolescent girls compared to older women and as such this population needs special attention [43].

Few surveyed facilities used immunologic and virologic criteria for transfer. Studies have shown that poor pre-transition outcomes are predictive of poor post-transition outcomes the utility of immunologic/virologic data in these contexts lie in the pre-transition identification of ALHIVs at high risk of poor outcomes in order to optimize their post-transition outcomes [40,44,45]. The clinical discretion of healthcare providers should be considered in the determination of transfer readiness. Providers may determine an ALHIV with, or at risk of immunologic/virologic failure not ready for transfer even when other milestones (including age) have been reached.

Support groups and routine disclosure procedures were reported as available at approximately a quarter and nearly half, respectively, of surveyed facilities. Psychosocial support and knowledge of HIV status have been reported to facilitate better treatment outcomes in some studies among ALHIV [46,47]. In a recent study, Nigerian ALHIV reported “seeking social support” as a coping strategy for major psychological stress related to living with HIV [48]. PEPFAR supports this strategy, and recommended peer psychosocial support for pediatric/adolescent adherence and retention in its Nigeria country operational plan that was in effect during study implementation [49] and thereafter [50]. It would be important to formalize the disclosure process and expand access to group or individual psychosocial support for ALHIV within both specialized and family-centered care models in Nigeria. The availability of trained and competent psychosocial support staff (including peers) to augment clinical service delivery to ALHIV cannot be overstated. Our findings show a paucity of these staff at surveyed sites.

A potential limitation to the generalizability of these findings is that only PEPFAR-supported health facilities were included in the survey. Health facilities not supported by PEPFAR may not have access to the same resources, and care in those facilities might differ from those surveyed in our study. However the cross-zonal, nationwide scope of our survey and the majority representation of PEPFAR country support for HIV services still lend to the study’s generalizability. Another limitation is that of the comprehensiveness of facility profile data: while we collected information on healthcare worker cadres in pediatric/adult/general HIV clinics, we did not collect detailed information on the number of specialties and entire scope of clinical services available at surveyed facilities. This could have helped characterize the facilities better in relation to transfer/transition practices.

## Conclusion

This situational analysis of ALHIV healthcare transition/transfer practices across Nigeria demonstrates that routine practices vary widely, and current transition procedures are not comprehensive. Implementation research is sorely needed to identify ALHIV transfer/transition models and strategies that are most feasible and effective for Nigeria’s largely non-specialized, family-centered HIV care settings. Approaches to determining transition readiness should not be limited to chronologic age; immunologic/virologic and mental health status should also be considered. Nigeria and similar high HIV burden, resource-constrained countries require evidence-informed public health approaches to consolidate gains made in HIV treatment among children by ensuring optimal transition/transfer outcomes for adolescents.

## Supporting information

S1 File Questionnaire for the ALHIV healthcare transition survey in Nigeria. (PDF)

## References

[1] UNAIDS. Global Report: UNAIDS report on the global AIDS epidemic 2013 [Internet]. UNAIDS. 2013. 198 p. Available from: http://files.unaids.org/en/media/unaids/contentassets/documents/epidemiology/2013/gr2013/UNAIDS_Global_Report_2013_en.pdf

[2] Kasedde S, Luo C, McClure C, Chandan U. Reducing HIV and AIDS in adolescents: opportunities and challenges. Curr HIV/AIDS Rep. 2013 6;10 (2):159–68. doi: 10.1007/s11904-013-0159-7 23563990 10.1007/s11904-013-0159-7

[3] UNICEF. Global and Regional Trends—UNICEF DATA [Internet]. 2016 [cited 2017 Aug 8]. Available from: http://data.unicef.org/topic/hivaids/global-regional-trends/

[4] World Health Organization. Health for the World’s Adolescents: A second chance in the second decade [Internet]. 2014 [cited 2017 Aug 8]. Available from: www.who.int/adolescent/second-decade

[5] UNICEF. Children, Adolescents and AIDS [Internet]. 2014 [cited 2017 Jul 14]. Available from: https://www.unicef.ch/sites/default/files/attachments/unicef_pb_statistical_update_on_children_adolescents_and_aids_2014.pdf

[6] Auld AF, Agolory SG, Shiraishi RW, Wabwire-Mangen F, Kwesigabo G, Mulenga M, et al Antiretroviral therapy enrollment characteristics and outcomes among HIV-infected adolescents and young adults compared with older adults—seven African countries, 2004–2013. Vol. 63, Morbidity and Mortality Weekly Report. 2014 p. 1097–103. 25426651 PMC5779521

[7] Ekouevi DK, Balestre E, Ba-Gomis FO, Eholie SP, Maiga M, Amani-Bosse C, et al Low retention of HIV-infected patients on antiretroviral therapy in 11 clinical centres in West Africa. Trop Med Int Heal. 2010;15(SUPPL. 1):34–42. 10.1111/j.1365-3156.2010.02505.x PMC2919326 20586958

[8] Agwu L. A, Lee L, Fleishman A. J, Voss C, Yehia R. B, Althoff N. K, et al Aging and Loss to Follow-up Among Youth Living With Human Immunodeficiency Virus in the HIV Research Network. J Adolesc Heal [Internet]. 2015;56(3):345–51. Available from: http://search.ebscohost.com/login.aspx?direct=true&db=cin20&AN=2012901378&site=ehost-live 10.1016/j.jadohealth.2014.11.009 PMC4378241 25703322

[9] Ryscavage P, Anderson EJ, Sutton SH, Reddy S, Taiwo B. Clinical outcomes of adolescents and young adults in adult HIV care. J Acquir Immune Defic Syndr [Internet]. 2011;58(2):193–7. Available from: http://www.ncbi.nlm.nih.gov/pubmed/21826014 doi: 10.1097/QAI.0b013e31822d7564 21826014 10.1097/QAI.0b013e31822d7564

[10] Fish R, Judd A, Jungmann E, O’Leary C, Foster C. Mortality in perinatally HIV-infected young people in England following transition to adult care: An HIV young persons’ network (HYPNet) audit. HIV Med. 2014;15(4):239–44. doi: 10.1111/hiv.12091 24112550 10.1111/hiv.12091

[11] Peter NG, Forke CM, Ginsburg KR, Schwarz DF, Onnorouille M, Lapointe N, et al Transition from pediatric to adult care: internists’ perspectives. Pediatrics [Internet]. 2009 2 1 [cited 2017 May 22];123(2):417–23. Available from: http://pediatrics.aappublications.org/cgi/doi/10.1542/peds.2008-0740 19171604 10.1542/peds.2008-0740

[12] Crowley R, Wolfe I, Lock K, McKee M. Improving the transition between paediatric and adult healthcare: a systematic review. Arch Dis Child. 2011 6;96(6):548–53. doi: 10.1136/adc.2010.202473 21388969 10.1136/adc.2010.202473

[13] Chu PY, Maslow GR, von Isenburg M, Chung RJ. Systematic Review of the Impact of Transition Interventions for Adolescents with Chronic Illness on Transfer From Pediatric to Adult Healthcare. J Pediatr Nurs [Internet]. 2015 9 [cited 2017 Aug 8];30(5):e19–27. Available from: http://linkinghub.elsevier.com/retrieve/pii/S0882596315002018 doi: 10.1016/j.pedn.2015.05.022 26209872 10.1016/j.pedn.2015.05.022 PMC4567416

[14] Davis AM, Brown RF, Taylor JL, Epstein RA, McPheeters ML. Transition Care for Children with Special Health Care Needs. Pediatrics [Internet]. 2014 11 12;134(5):900–8. Available from: http://www.ncbi.nlm.nih.gov/pmc/articles/PMC4533283/ doi: 10.1542/peds.2014-1909 25287460 10.1542/peds.2014-1909 PMC4533283

[15] Boudreau ME, Fisher CM. Providing Effective Medical and Case Management Services to HIV-Infected Youth Preparing to Transition to Adult Care. J Assoc Nurses AIDS Care [Internet]. 2017 7 10;23(4):318–28. Available from: http://dx.doi.org/10.1016/j.jana.2011.06.003 10.1016/j.jana.2011.06.003 21820326

[16] Maturo D, Powell A, Major-Wilson H, Sanchez K, De Santis JP, Friedman LB, et al Development of a protocol for transitioning adolescents with HIV infection to adult care. J Pediatr Health Care [Internet]. 2007 1 1 [cited 2017 Aug 8];25(1):16–23. Available from: http://www.ncbi.nlm.nih.gov/pubmed/21147403 10.1016/j.pedhc.2009.12.005 21147403

[17] Hussen SA, Chahroudi A, Boylan A, Camacho-Gonzalez AF, Hackett S, Chakraborty R. Transition of youth living with HIV from pediatric to adult-oriented healthcare: a review of the literature. Future Virol [Internet]. 2014 10 [cited 2017 Aug 8];9(10):921–9. Available from: http://www.ncbi.nlm.nih.gov/pubmed/25983853 10.2217/fvl.14.73 PMC4433446 25983853

[18] Dahourou DL, Gautier-Lafaye C, Teasdale CA, Renner L, Yotebieng M, Desmonde S, et al Transition from paediatric to adult care of adolescents living with HIV in sub-Saharan Africa: challenges, youth-friendly models, and outcomes. J Int AIDS Soc [Internet]. 2017;20(0):34–49. Available from: http://www.jiasociety.org/index.php/jias/article/view/21528 10.7448/IAS.20.4.21528 PMC5577723 28530039

[19] UNAIDS. UNAIDS data 2017 [Internet]. 2017 [cited 2018 Feb 2]. Available from: http://www.unaids.org/sites/default/files/media_asset/20170720_Data_book_2017_en.pdf

[20] UNAIDS. HIV indicators [Internet]. AIDSinfo 2016 Data. 2017 [cited 2017 Jan 1]. Available from: http://aidsinfo.unaids.org/

[21] Slogrove AL, Mahy M, Armstrong A, Davies M-A. Living and dying to be counted: What we know about the epidemiology of the global adolescent HIV epidemic. J Int AIDS Soc [Internet]. 2017 4 10 [cited 2018 Feb 19];20(0):21520 Available from: http://www.ncbi.nlm.nih.gov/pubmed/28530036 doi: 10.7448/IAS.20.4.21520 28530036 10.7448/IAS.20.4.21520 PMC5719718

[22] Federal Ministry of Health Nigeria. Clinical Protocol for the Health and Development of Adolescent & Young People in Nigeria [Internet]. 2011 [cited 2017 May 22]. Available from: http://www.health.gov.ng/doc/ClinicalProtocol.pdf

[23] Federal Ministry of Health Nigeria/UNFPA. Action Plan for Advancing Young People’s Health & Development in Nigeria: 2010 ‐ 2012. 2010. 1–44 p.

[24] Ezeanolue EE, Powell BJ, Patel D, Olutola A, Obiefune M, Dakum P, et al Nigeria Implementation Science Alliance. Identifying and Prioritizing Implementation Barriers, Gaps, and Strategies through the Nigeria Implementation Science Alliance: Getting to Zero in the Prevention of Mother-to-Child Transmission of HIV. J Acquir Immune Defic Syndr [Internet]. 2016;72 Suppl 2:S161–6. Available from: http://www.ncbi.nlm.nih.gov/pubmed/27355504 27355504 10.1097/QAI.0000000000001066 PMC5113249

[25] Federal Ministry of Health Nigeria. National Guidelines for HIV and AIDS Treatment and care in adolescents and adults. Abuja; 2010.

[26] Olakunde BO, Ndukwe CD. Improved Domestic Funding Enhances the Sustainability of HIV/AIDS Response in Nigeria. Ann Glob Heal [Internet]. 2015 9 [cited 2018 Feb 19];81(5):684–8. Available from: http://linkinghub.elsevier.com/retrieve/pii/S2214999615012655 10.1016/j.aogh.2015.10.005 27036726

[27] PEPFAR Dashboards [Internet]. [cited 2018 Feb 19]. Available from: https://data.pepfar.net/country/expenditure?country=Nigeria

[28] Federal Government of Nigeria National AIDS Spending Assessment (NASA) for the Period: 2013–2014 Level and Flow of Resources and Expenditures of the National HIV and AIDS Response [Internet]. [cited 2018 Feb 19]. Available from: http://www.unaids.org/sites/default/files/media/documents/Nigeria_NASA_2013.pdf

[29] Sam-Agudu NA, Pharr JR, Bruno T, Cross CL, Cornelius LJ, Okonkwo P, et al Adolescent Coordinated Transition (ACT) to improve health outcomes among young people living with HIV in Nigeria: Study protocol for a randomized controlled trial. Trials. 2017;18(1). 10.1186/s13063-017-2347-z PMC5729403 29237487

[30] National Guidelines for HIV Prevention, Treatment and Care [Internet]. [cited 2018 Feb 19]. Available from: https://aidsfree.usaid.gov/sites/default/files/ng_national_guidelines_hiv.pdf

[31] Kennedy A, Sawyer S. Transition from pediatric to adult services: are we getting it right? Curr Opin Pediatr [Internet]. 2008 8 [cited 2018 Feb 19];20(4):403–9. Available from: http://www.ncbi.nlm.nih.gov/pubmed/18622194 doi: 10.1097/MOP.0b013e328305e128 18622194 10.1097/MOP.0b013e328305e128

[32] Got Transition Center for Health Care Transition. Six Core Elements of Health Care Transition 2.0 [Internet]. 2014 [cited 2017 Aug 8]. Available from: http://www.gottransition.org/resourceGet.cfm?id=206.

[33] World Health Organization. HIV and Adolescents from Guidance to Action [Internet]. [cited 2017 Aug 8]. Available from: http://apps.who.int/adolescent/hiv-testing-treatment/page/Transition

[34] IBM SPSS Statistics for Windows. Armonk, NY: IBM Corp; 2012.

[35] Sohn AH, Vreeman RC, Judd A. Tracking the transition of adolescents into adult HIV care: a global assessment. J Int AIDS Soc [Internet]. 2017;20(0):19–21. Available from: http://www.jiasociety.org/index.php/jias/article/view/21878 10.7448/IAS.20.4.21878 PMC5577733 28530035

[36] Kung TH, Wallace ML, Snyder KL, Robson VK, Mabud TS, Kalombo CD, et al South African healthcare provider perspectives on transitioning adolescents into adult HIV care. S Afr Med J [Internet]. 2016 7 6 [cited 2017 May 22];106(8):804–8. Available from: http://www.ncbi.nlm.nih.gov/pubmed/27499409 doi: 10.7196/SAMJ.2016.v106i8.10496 27499409 10.7196/SAMJ.2016.v106i8.10496

[37] Katusiime C, Parkes-Ratanshi R, Kambugu A. Transitioning behaviourally infected HIVpositive young people into adult care: Experiences from the young person’s point of view. South Afr J HIV Med [Internet]. 2013 [cited 2017 May 22];14(1):20–4. Available from: http://www.sajhivmed.org.za/index.php/hivmed/article/view/98/157

[38] Mark D, Armstrong A, Andrade C, Penazzato M, Hatane L, Taing L, et al HIV treatment and care services for adolescents: a situational analysis of 218 facilities in 23 sub-Saharan African countries. J Int AIDS Soc [Internet]. 2017 4 10 [cited 2018 Feb 19];20(0):21591 Available from: http://www.ncbi.nlm.nih.gov/pubmed/28530038 doi: 10.7448/IAS.20.4.21591 28530038 10.7448/IAS.20.4.21591 PMC5719719

[39] Lam PK, Fidler S, Foster C. A review of transition experiences in perinatally and behaviourally acquired HIV-1 infection; same, same but different? J Int AIDS Soc. 2017 5;20(Suppl 3):91–9. 10.7448/IAS.20.4.21506 PMC5577725 28530044

[40] Okoboi S, Ssali L, I Yansaneh A, Bakanda C, Birungi J, Nantume S, et al Factors associated with long-term antiretroviral therapy attrition among adolescents in rural Uganda: a retrospective study. J Int AIDS Soc [Internet]. 2016 7 20 [cited 2018 Feb 19];19(5 (Suppl 4)). Available from: http://doi.wiley.com/10.7448/IAS.19.5.20841 10.7448/IAS.19.5.20841 PMC4956735 27443271

[41] Pettitt ED, Greifinger RC, Phelps BR, Bowsky SJ. Improving health services for adolescents living with HIV in sub-Saharan Africa: a multi-country assessment. Afr J Reprod Health. 2013 12;17(4 Spec No):17–31. 24689314

[42] Davies M-A, Tsondai P, Tiffin N, Eley B, Rabie H, Euvrard J, et al Where do HIV-infected adolescents go after transfer?—Tracking transition/transfer of HIV-infected adolescents using linkage of cohort data to a health information system platform. J Int AIDS Soc [Internet]. 2017 4 16 [cited 2018 Feb 19];20(Suppl 3):21668 Available from: http://doi.wiley.com/10.7448/IAS.20.4.21668 28530037 10.7448/IAS.20.4.21668 PMC5577779

[43] Callahan T, Modi S, Swanson J, Ng’eno B, Broyles LN. Pregnant adolescents living with HIV: what we know, what we need to know, where we need to go. J Int AIDS Soc [Internet]. 2017 [cited 2018 Feb 19];20(1):21858 Available from: http://doi.wiley.com/10.7448/IAS.20.1.21858 28782334 10.7448/IAS.20.1.21858 PMC5577684

[44] Weijsenfeld AM, Smit C, Cohen S, Wit FWNM, Mutschelknauss M, van der Knaap LC, et al Virological and Social Outcomes of HIV-Infected Adolescents and Young Adults in The Netherlands Before and After Transition to Adult Care. Clin Infect Dis [Internet]. 2016 10 15 [cited 2018 Feb 19];63(8):1105–12. Available from: https://academic.oup.com/cid/article-lookup/doi/10.1093/cid/ciw487 27439528 10.1093/cid/ciw487

[45] Sainz T, Fernandez Mcphee C, Jimenez De Ory S, Gonzalez-Tome M, Rubio R, Bernardino J. Transition to adult units: situation and evolution of vertically HIV infected youths in Spain. In: Conference on Retroviruses and Opportunistic Infections (CROI). 2015. p. 918.

[46] Ridgeway K, Dulli LS, Murray KR, Silverstein H, Dal Santo L, Olsen P, et al Interventions to improve antiretroviral therapy adherence among adolescents in low- and middle-income countries: A systematic review of the literature. Paraskevis D, editor. PLoS One [Internet]. 2018 1 2 [cited 2018 Feb 19];13(1):e0189770 Available from: http://dx.plos.org/10.1371/journal.pone.0189770 29293523 10.1371/journal.pone.0189770 PMC5749726

[47] Vreeman RC, Gramelspacher AM, Gisore PO, Scanlon ML, Nyandiko WM. Disclosure of HIV status to children in resource-limited settings: a systematic review. J Int AIDS Soc [Internet]. 2013 5 27 [cited 2018 Feb 19];16:18466 Available from: http://www.ncbi.nlm.nih.gov/pubmed/23714198 doi: 10.7448/IAS.16.1.18466 23714198 10.7448/IAS.16.1.18466 PMC3665848

[48] Folayan MO, Cáceres CF, Sam-Agudu NA, Odetoyinbo M, Stockman JK, Harrison A. Psychological Stressors and Coping Strategies Used by Adolescents Living with and Not Living with HIV Infection in Nigeria. AIDS Behav [Internet]. 2017 9 7 [cited 2018 Feb 19];21(9):2736–45. Available from: http://www.ncbi.nlm.nih.gov/pubmed/27605363. doi: 10.1007/s10461-016-1534-3 27605363 10.1007/s10461-016-1534-3 PMC6510022

[49] United States President’s Emergency Plan for AIDS Relief. Nigeria Country Operational Plan (COP) 2016 Strategic Direction Summary. [cited 2018 May 11]. Available from: https://www.pepfar.gov/documents/organization/257635.pdf

[50] United States President’s Emergency Plan for AIDS Relief. Nigeria Country Operational Plan (COP) 2017 Strategic Direction Summary. [cited 2018 May 11]. Available from: https://ng.usembassy.gov/wp-content/uploads/sites/177/2017/03/2017StrategicDirectionSummary_Draft6_03-28-2017.pdf

