# Supplementary material for: Pediatric to adult healthcare transitioning for adolescents living with HIV in Nigeria: A national survey
Source: PLoS One. 2018 Jun 12;13(6):e0198802. doi: 10.1371/journal.pone.0198802 (PMC5997346; doi:10.1371/journal.pone.0198802)
Supplement: S1 File — (PDF) [file pone.0198802.s001.pdf]

SECTION A: SITE PROFILE

Facility Name: ____

Level of Comprehensive Care: ☐ Tertiary ☐ Secondary

State: ________________________ **IP:** ________________________

	10-14 years		15-19 years	
ALHIV PATIENT DATA	**Male**	**Female**	**Male**	**Female**
**No. of ALHIV (10-19 year olds) currently enrolled at facility:**				
**No. of ALHIV (10-19 year olds) currently on ART at facility:**				

SECTION B: CLINIC SERVICES

1. **How long has the facility been providing adult HIV services?**
   ☐ Less than 1 year
   ☐ 1-3 years
   ☐ 4-6 years
   ☐ More than 6 years
   ☐ Don't Know
   ☐ N/A (not applicable)

2. **On average, how many health care workers provide adult HIV services?**

Doctors	Nurses

3. **What other types of healthcare workers provide adult HIV services at this facility? (Specify type and number)**

4. **Does the facility provide pediatric HIV services?** ☐ Yes ☐ No <u>**(If NO, END SURVEY here)**</u>

5. **If yes to Q4, how long has the facility been providing paediatric HIV services?**
   ☐ Less than 1 year
   ☐ 1 -3 years
   ☐ 4-6 years
   ☐ More than 6 years
   ☐ Don't Know
   ☐ N/A

6. **Is there a pediatric HIV clinic at the facility (dedicated time and space for children)?** ☐ Yes ☐ No

7. **Are the adult HIV care providers the same people who provide paediatric HIV care? (If Yes, skip to Section C)** ☐ Yes ☐ No ☐ N/A

8. **If no to Q7, on average, how many healthcare workers provide paediatric HIV services? (If N/A, leave blank)**

Doctors	Nurses

9. **What other types of healthcare workers provide pediatric HIV services at this facility? (Specify type and number).**

SECTION C: ADOLESCENT CARE SERVICES

10. **Is there an adolescent HIV clinic at the facility (a dedicated time and space for ALHIV)?** ☐ Yes ☐ No
11. **Have clinic staff received training to work with adolescents, teenagers or youth? (Choose only one answer).** ☐ All ☐ None ☐ Some ☐ Not sure
12. **Is there a standard practice among providers for helping parents to disclose HIV status to their adolescents?** ☐ Yes ☐ No
13. **If yes to Q12, is there a written protocol (If yes, please provide a copy)?** ☐ Yes ☐ No
14. **At what specific age or age range do providers encourage parents to start the disclosure process to an HIV-infected child?** Age/range___________ ☐ N/A

SECTION D: ADOLESCENT SUPPORT GROUP SERVICES

15. **Does the facility hold dedicated support groups for ALHIV?** ☐ Yes ☐ No (Skip to section E)
16. **If yes to Q15, at what age/age range do you recommend the support groups to patients?** Age: ______________________
17. **If support groups are divided by age, please specify age(s):** ______________________________
18. **Are there separate support groups for females and males?** ☐ Yes ☐ No
19. **How often do the adolescent support groups meet?**
    - ☐ Daily
    - ☐ Weekly
    - ☐ Monthly
    - ☐ Every 2-3 months
    - ☐ Irregularly
    - ☐ Other (specify)_____________
20. **What is the average number of ALHIV attending the support group meetings?** No.____________
21. **When do support group meetings take place?**
    - ☐ Before scheduled clinic hours
    - ☐ After scheduled clinic hours
    - ☐ On a day other than clinic day- weekday
    - ☐ On a day other than clinic day - weekend
    - ☐ When patients decide
    - ☐ Other (specify)
22. **What is the duration of the support group meetings in hours or minutes?** _________________
23. **Where do support group meetings take place (check all that apply)?**
    - ☐ Physically within clinic, or hospital grounds
    - ☐ At designated location within the community
    - ☐ On social media (specify)___________________________
    - ☐ Other venue (specify)_____________________________
24. **Who participates in the support groups?**
    - ☐ ALHIV only
    - ☐ ALHIV + Others (e.g. siblings of ALHIV, other adolescents)
25. **Who facilitates the adolescent support group meetings (check all that apply)?**
    - ☐ Peer (Adolescent/Youth) Facilitator
    - ☐ CHEW / CHO
    - ☐ Other Healthcare Worker (nurse, nurse midwife, doctor)
    - ☐ Other adult (non-healthcare provider)
    - ☐ Other (specify) _____________________________________
    - ☐ None (group members do this on their own)

SECTION E: TRANSFER OF ADOLESCENTS FROM PEDIATRIC TO ADULT CARE

26. **Is there a standard practice for transferring ALHIV to the adult clinic?** ☐ Yes ☐ No ☐ ALHIV are not transferred (Adult, Child, ALHIV mixed clinic)
If ALHIV not transferred, STOP SURVEY HERE

27. **If yes to Q26, is there a written protocol for transferring ALHIV to adult clinic? (If yes, please provide a copy)** ☐ Yes ☐ No ☐ N/A

28. **If yes to Q26, which of the following criteria are used to determine that it is time for transfer? (please check all that apply):**

a.	**Age**	☐ Yes ☐ No	b.	**Patient aware of diagnosis**	☐ Yes ☐ No
c.	**Patient has CD4 count ≥ 500**	☐ Yes ☐ No	d.	**Readiness assessment**	☐ Yes ☐ No
e.	**Viral load**	☐ Yes ☐ No	g.	**Marriage**	☐ Yes ☐ No
f.	**Demonstrated knowledge and understanding about HIV, adherence, drugs**	☐ Yes ☐ No	h.	**Pregnancy**	☐ Yes ☐ No
			i.	**Other (describe):**	

29. **At what age (years) are adolescents transferred from the pediatric clinic to the adult clinic?** ☐ 15 ☐ 16 ☐ 17 ☐ 18 ☐ 19 ☐ Other age__________ ☐ N/A

30. **To where does the transfer occur?** ☐ Within same facility ☐ Referral to outside facility

31. **At the time of transfer, is there formal communication between pediatric and adult clinic physicians/nurses?** ☐ Yes ☐ No

32. **If yes to Q31, what form of communication is it?** ☐ Verbal ☐ Written ☐ Both

33. **Is there any post-transfer follow-up of the ALHIV by pediatric clinic staff?** ☐ Yes ☐ No

34. **How does this follow-up occur?** ☐ Contact ALHIV/parent ☐ Contact Adult Clinic
☐ Other (specify):____________________

END OF SURVEY.
